# Supplementary material for: Distinct functions for the paralogous RBM41 and U11/U12-65K proteins in the minor spliceosome
Source: Nucleic Acids Res. 2024 Mar 18;52(7):4037–52. doi: 10.1093/nar/gkae070 (PMC11039992; doi:10.1093/nar/gkae070)
Supplement: gkae070_Supplemental_Files [file gkae070_supplemental_files.zip › Supplementary figures.pdf]

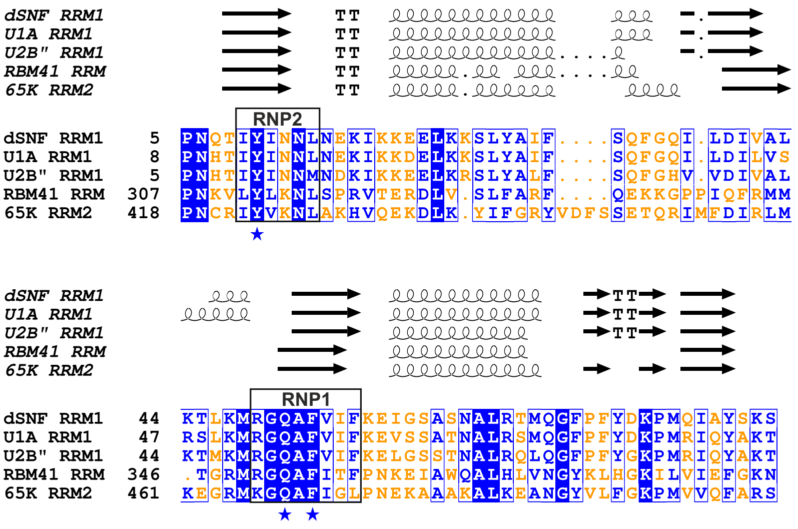

Supplementary Figure 1. Multiple sequence alignment of human RBM41, U11/U12-65K, U1A, U2B″ and *Drosophila melanogaster* SNF (sans fille) RNA recognition motifs. Alignment was carried out using MAFFT and visualized using ESPript 3.0. Protein secondary structure information was extracted from the following PDB structures: 6F4I, 1NU4, 1A9N, 2CPX, 5OBN. The conserved YQF triad residues are indicated with blue stars.

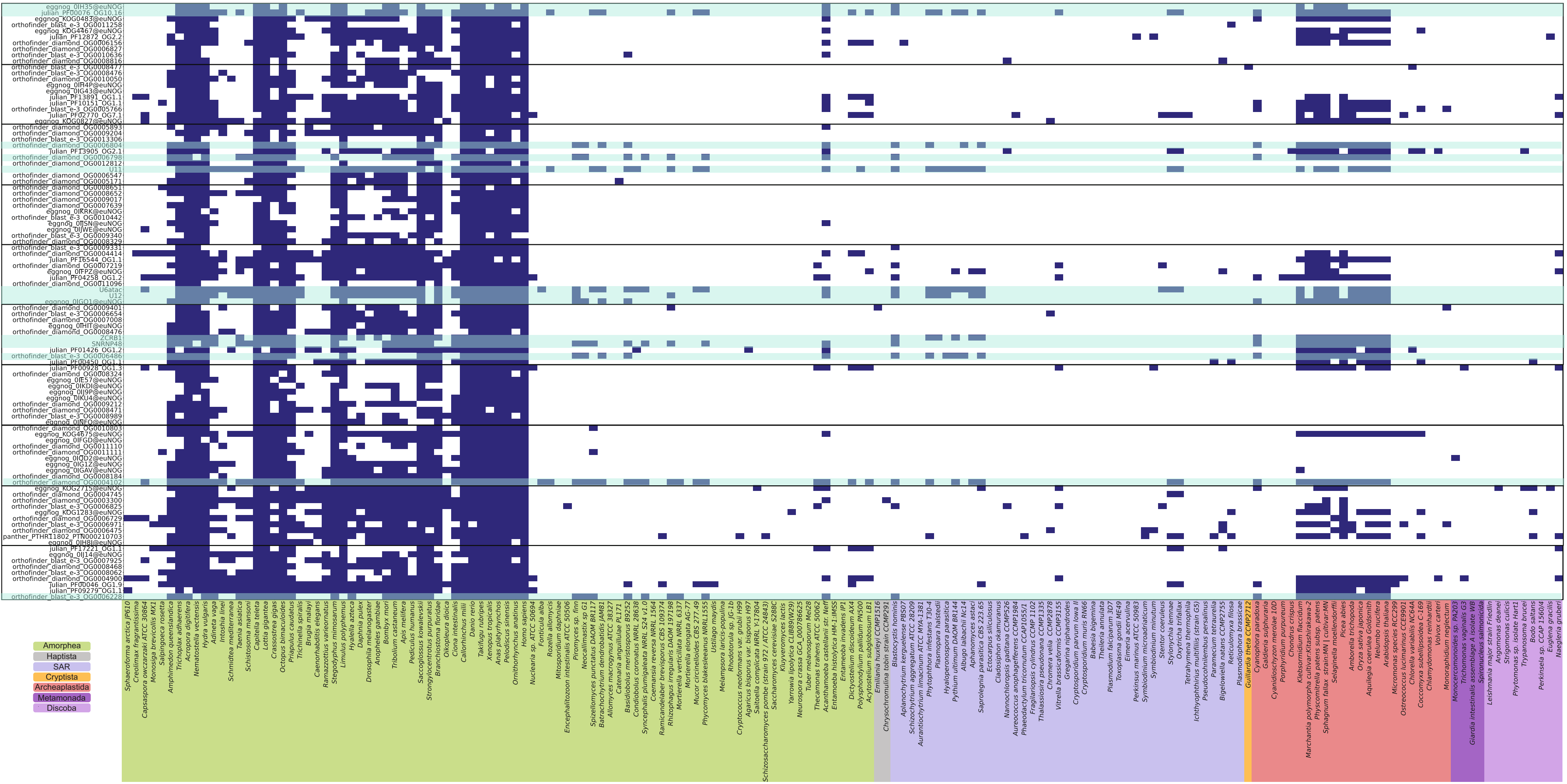

Supplementary Figure 2. Orthologous groups coevolving with *RBM41.* Orthologous groups represent those listed in the Supplementary Table 3. Cyan shading indicate orthologus groups composed of known minor spliceosome components.

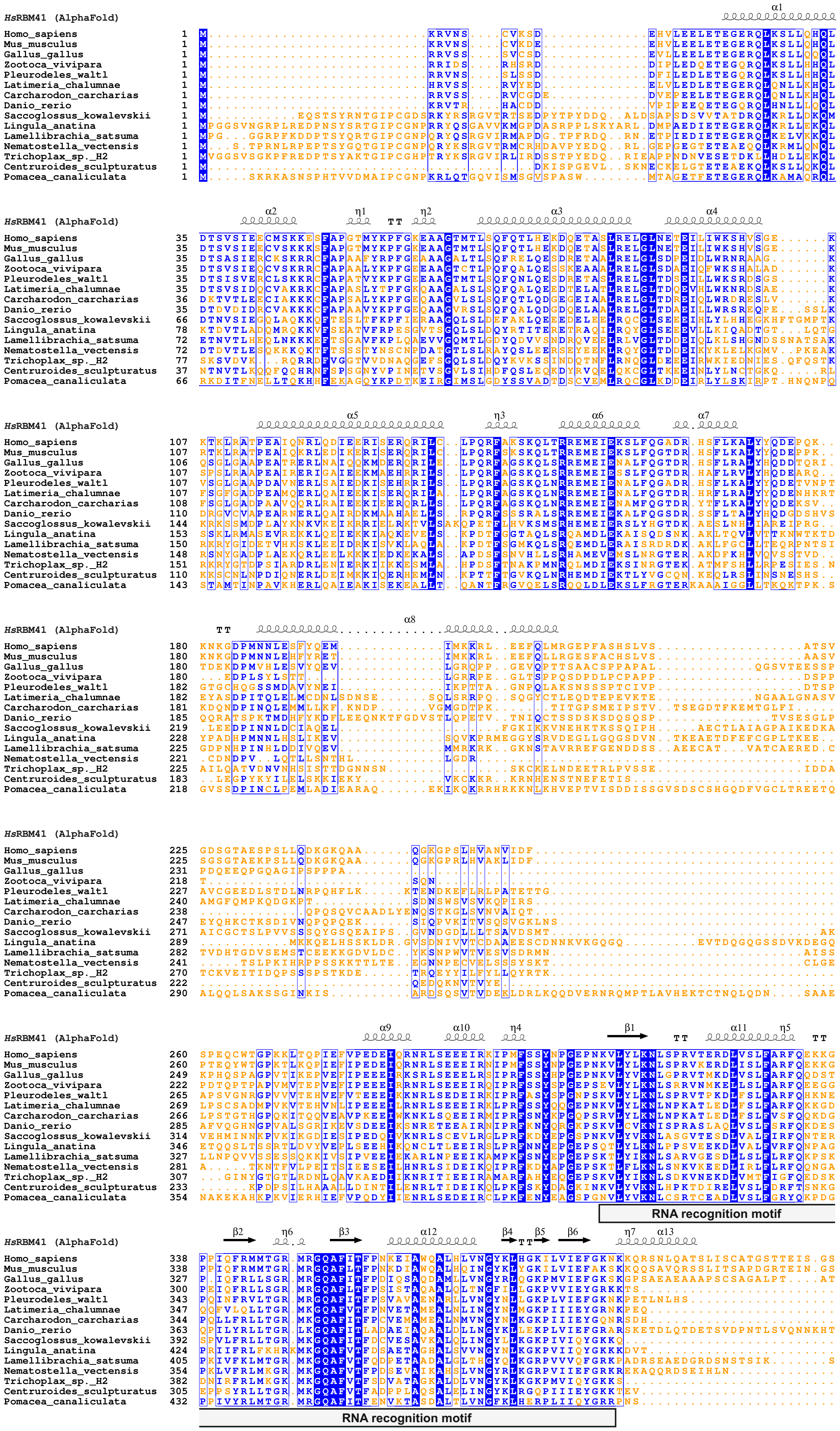

Supplementary Figure 3. Multiple sequence alignment of RBM41 orthologs from 15 animal species. Alignment was carried out using MAFFT and visualized using ESPript 3.0. Secondary structures extracted from AlphaFold-predicted human RBM41 structure (Figure 1D) are shown. Sequences used for the alignment are listed in Supplementary Table 2.

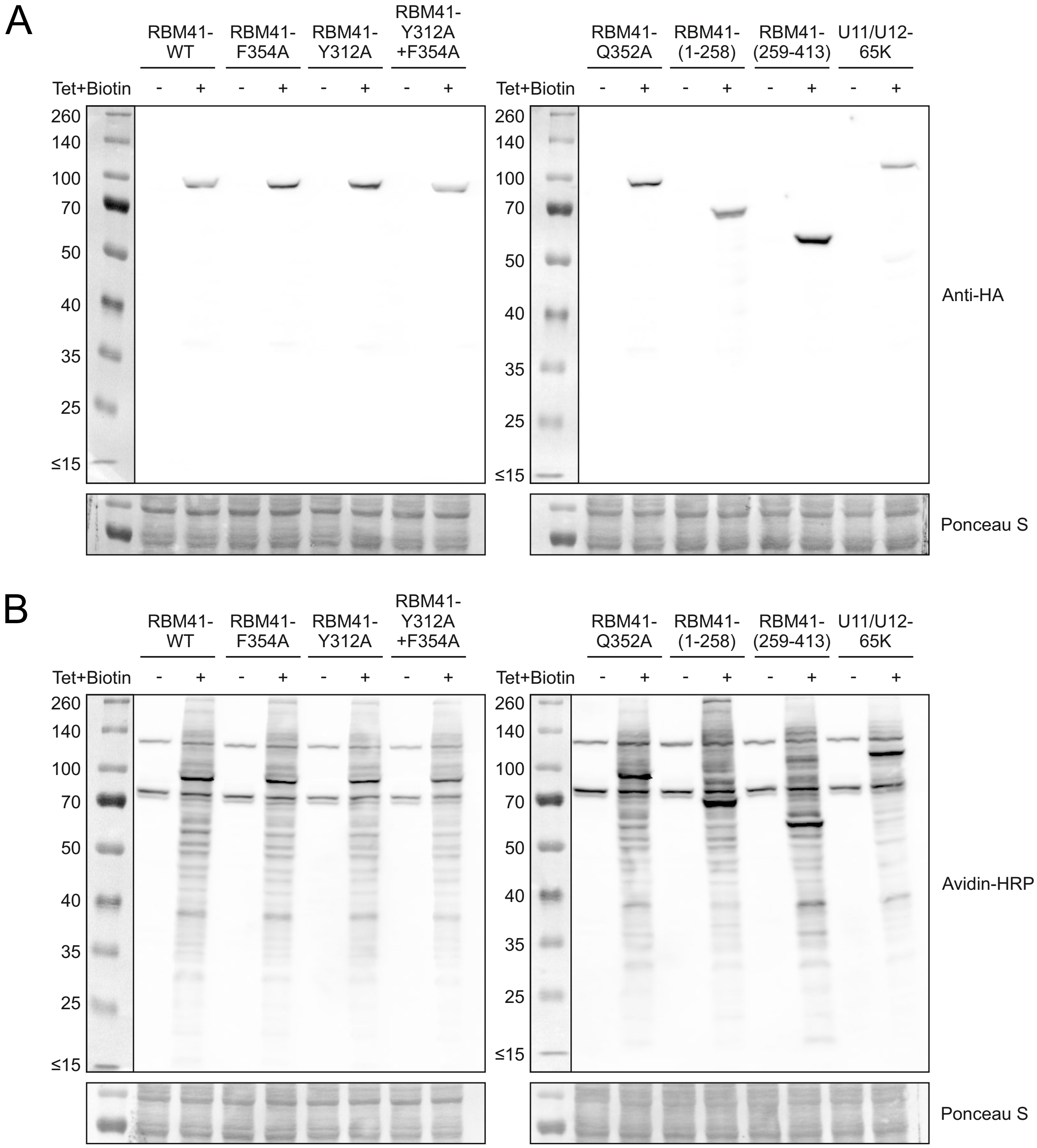

Supplementary Figure 4. Validation of Flp-in 293 cell lines used in BioID experiments. MAC-tagged protein expression and biotinylation was induced by addition of tetracycline and biotin for 24 hours before harvesting of cells.
(A) Induction of MAC-tagged proteins detected by western blot with anti-HA antibody.
(B) Biotinylation detected by western blot with avidin-HRP.

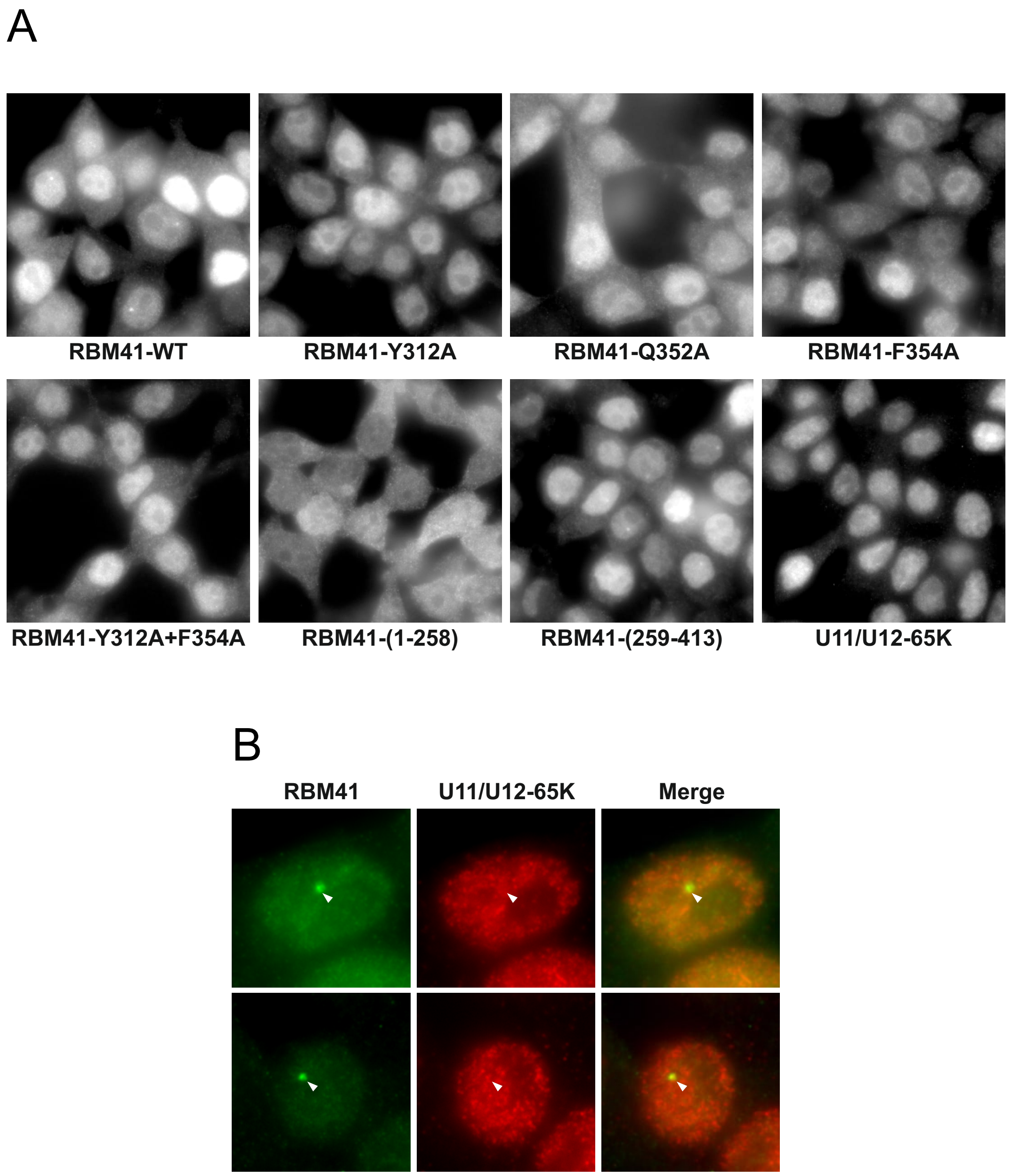

Supplementary Figure 5.
(A) Localization MAC-tagged constructs in Flp-in 293 cell lines. Expression was induced with tetracycline for 24 h and immunofluorescence carried out with anti-HA antibody.
(B) Immunofluorescence staining of HEK293 cells for with anti-U11/U12-65K and anti-RBM41 antibodies.

```
Protospacer
PAM site
```

Guide RNA targeting *RBM41* exon 2

```
WT   GGGGAGAGGCAGCTGAAAAGCCTCCTTCAGCATCAACTTGATACTTCTGTCTCCATTGAGGAGT

C9   GGGGAGAGGCAGCTGAAAAGCCTCCTTTCAGCATCAACTTGATACTTCTGTCTCCATTGAGGAG ins 1 bp
     GGGGAG-------------------TTCAGCATCAACTTGATACTTCTGTCTCCATTGAGGAGT del 19 bp
```

1 bp insertion and 19 bp deletion confirmed by DECODR, no additional variants detected

```
C10  GGGGAGAGGCAGCTGAAAAGCCTCCTGTCAGCATCAACTTGATACTTCTGTCTCCATTGAGGAG ins 1 bp
     GGGGAGAGGCAGCTGAAAAGCCTCCTTATCAGCATCAACTTGATACTTCTGTCTCCATTGAGGA ins 2 bp
     GGGGAGAGGCAGCTGAAAAGCCTCCT------------TGATACTTCTGTCTCCATTGAGGAGT del 12 bp
     GGGGAGAGGCAGCTGAAAAGCCTCCT-----CATCAACTGATACTTCTGTCTCCATTGAGGAGT del 4 bp
```

12 bp deletion and both insertions confirmed by DECODR, additional 4 bp deletion detected

```
C11  GGGGAGAGGCAGCTGAAAAGCCTCCT--ATCATCAACTTGATACTTCTGTCTCCATTGAGGAGT del 2 bp+G>T subst.
     GGGGAGAGGCAGCTGAAAAGCC-----------------------TCTGTCTCCATTGAGGAGT del 23 bp
```

2 bp deletion confirmed and additional 23 bp deletion detected by DECODR

2 bp and 23 bp deletions confirmed by RNA-seq

Guide RNA targeting *RBM41* exon 3

```
WT   GAGAGCTTTGCTCCTGGTACTATGTACAAGCCCTTTGGGAAGGAAGCAGCTGGGACTATGACT

D1   GAGAGCTTTGCTCCTGGTACTATGTACAAGCCCTT--GGAAGGAAGCAGCTGGGACTATGACT  del 2 bp
     GAGAGCTTTGCTCCTGGTACTATGTACAAGCCCT--------GAAGCAGCTGGGACTATGACT  del 8 bp
     GAGAGCTTTGCTCCTGGTACTATG--------------GAAGGAAGCAGCTGGGACTATGACT  del 14 bp
```

2 bp, 8 bp and 14 bp deletions confirmed by DECODR, no additional variants detected

2 bp and 8 bp deletions confirmed by RNA-seq, but no reads supporting 14 bp deletion

```
D4   GAGAGCTTTGCTCCTGGTACTATGTACAAGCCCT----------------TGGGACTATGACT  del 16 bp
     GAGAGCTTTGCTCCTGGTACTATGTACAAGCCCTTTGCTTTGGGAAGGAAGCAGCTGGGACTA  dup 5 bp
     GAGAGCTTTGCTCCTGGTACTATG-------------GGAAGGAAGCAGCTGGGACTATGACT  del 13 bp
```

16 bp and 13 bp deletions and 5 bp duplication confirmed by DECODR, no additional variants detected

```
D14  GAGAGCTTTGCTCCTGGTACTATGTACAAGCCCTT--GGAAGGAAGCAGCTGGGACTATGACT  del 2 bp
     GAGAGCTTTGCTCCTGGTACTATGTACAAGCCCTTTG-------AGCAGCTGGGACTATGACT  del 7 bp
     GAGAGCTTTGCTCCTGGTACTATGTACAAGCCCTTTGG-AAGGAAGCAGCTGGGACTATGACT  del 1 bp
```

2 bp, 7 bp and 1 bp deletions confirmed by DECODR

2 bp, 7 bp and 1 bp deletions confirmed by RNA-seq

Supplementary Figure 6. The sequeces of individual HEK293 the RBM41 Knock-Out clones. The targets of the guide RNAs in RBM41 exons 2 and 3 are indicated in the WT sequece. Clones C11, D1, and D14 were used in the RNAseq analysis. C9, C10, and D4 were used in RT-PCR validation experiments.
